# Multi-carbohydrase complex supplementation improves crude protein and energy utilization of corn-soybean-based diets with or without HP-DDGS in growing pigs

**DOI:** 10.1093/tas/txag025

**Published:** 2026-02-28

**Authors:** Stephane Alverina Briguente da Motta, Wagner Azis Garcia de Araújo, Naiara Simarro Fagundes, Adriana Berti Toscan, Afonso Luna Miranda, Alejandra Gutierrez Riaño, Giovana Thais Soares Pereira, Raphaela Ribeiro Neves, Geovana da Silva Ribeiro, Bruno Alexander Nunes Silva

**Affiliations:** Institute of Agricultural Sciences/ICA, Universidade Federal de Minas Gerais (UFMG), Montes Claros, Minas Gerais 39404-547, Brazil; Animal Science Departament, Universidade Federal de Lavras (UFLA), Lavras, Minas Gerais 37200-900, Brazil; Animal Science Unit, Instituto Federal de Educação, Ciência e Tecnologia Norte de Minas Gerais (IFNMG), Januária, Minas Gerais 39480-000, Brazil; Adisseo Brasil, São Paulo, SP 05804-900, Brazil; Adisseo Brasil, São Paulo, SP 05804-900, Brazil; Institute of Agricultural Sciences/ICA, Universidade Federal de Minas Gerais (UFMG), Montes Claros, Minas Gerais 39404-547, Brazil; Animal Science Departament, Universidade Federal de Lavras (UFLA), Lavras, Minas Gerais 37200-900, Brazil; Institute of Agricultural Sciences/ICA, Universidade Federal de Minas Gerais (UFMG), Montes Claros, Minas Gerais 39404-547, Brazil; Animal Science Departament, Universidade Federal de Lavras (UFLA), Lavras, Minas Gerais 37200-900, Brazil; Institute of Agricultural Sciences/ICA, Universidade Federal de Minas Gerais (UFMG), Montes Claros, Minas Gerais 39404-547, Brazil; Animal Science Departament, Universidade Federal de Lavras (UFLA), Lavras, Minas Gerais 37200-900, Brazil; Institute of Agricultural Sciences/ICA, Universidade Federal de Minas Gerais (UFMG), Montes Claros, Minas Gerais 39404-547, Brazil; Institute of Agricultural Sciences/ICA, Universidade Federal de Minas Gerais (UFMG), Montes Claros, Minas Gerais 39404-547, Brazil; Institute of Agricultural Sciences/ICA, Universidade Federal de Minas Gerais (UFMG), Montes Claros, Minas Gerais 39404-547, Brazil; Animal Science Departament, Universidade Federal de Lavras (UFLA), Lavras, Minas Gerais 37200-900, Brazil

**Keywords:** carbohydrase, digestibility, enzymes, DDGS, pigs

## Abstract

The use of high protein corn dried distillers’ grains with solubles (HP-DDGS) presents distinct nutritional and economic value compared to corn and soybean meal. This co-product is characterized by a high concentration of non-starch polysaccharides. The hypothesis of this study was that the inclusion of a multi-carbohydrase complex (MCC) in a corn-soybean based diet with or without HP-DDGS would improve nitrogen and energy digestibility and utilization in growing pigs. A total of 8 pigs were used in a digestibility study. Animals were assigned to 4 dietary treatments using a replicated 4 × 4 Latin square design. Experimental diets consisted of two basal diets: a corn–soybean meal diet and a corn–soybean meal diet containing 20% HP-DDGS. Each basal diet was formulated with or without the inclusion of MCC. Data were analyzed using an ANOVA with HP-DDGS inclusion, enzyme inclusion, animal, and period as the main effects. Means were then compared by the Student Newman-keuls test. The inclusion of HP-DDGS reduced (*P* < 0.001) the apparent total tract digestibility of dry matter, crude protein, gross energy, energy metabolizability coefficient, and decreased digestible energy and metabolizable energy compared to the corn-soybean diet. Conversely, MCC supplementation improved (*P* < 0.05) nutrient and energy digestibility, regardless of diet composition. The present study demonstrated that the use of a MCC in growing pig diets has the potential to improve nutrient digestibility and utilization not only when HP-DDGS is used, but also in regular corn-soybean based diets.

## Graphical Abstract

**Graphical Abstract:**
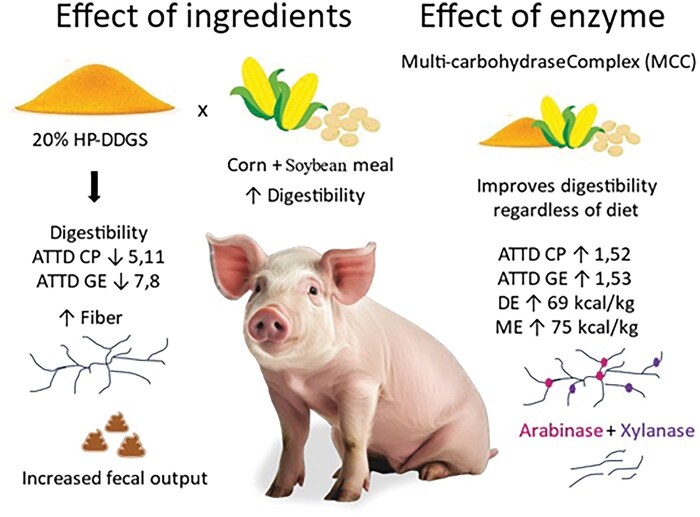

## Teaser

The inclusion of a multi-carbohydrase complex in pig diets significantly improved nutrient digestibility in both conventional corn- and soybean-based diets and HP-DDGS–containing diets, highlighting the importance of enzyme supplementation. These results indicate that multi-carbohydrase complex is a promising strategy to optimize nutrient utilization and enable the sustainable and cost-effective inclusion of ethanol by-products in swine nutrition.

## Introduction

The diet of pigs in Brazil is mainly formulated based on corn and soybean meal, which have significant price variability throughout the year, often making production economically instable. Feed costs for pigs represent approximately 80% of total production costs, increasing the pressure to introduce alternative feed ingredients in animal nutrition (Da Silva et al. 2015).

In Brazil, the large-scale production of corn has led to a growing trend of using it for ethanol production. During this process, the grain is refined, generating several co-products, including high-protein dried distillers’ grains with solubles (HP-DDGS). HP-DDGS contains high concentrations of crude protein (CP), typically ranging from 37 to 40% (Rho et al. 2017; Espinosa and Stein 2018; Da Motta et al. 2024). In addition, it provides elevated levels of fat and digestible phosphorus, making it a promising ingredient for the partial replacement of corn and soybean meal in animal nutrition. Additionally, it provides a sustainable way to direct this by-product from the ethanol industry (Wu et al. 2016). It is important to note that during the ethanol distillation process, most of the starch in the grain is converted into ethanol, while the fiber remains unused. As a result, DDGS contains less starch and a higher fiber content compared to its original raw material.

Non-starch polysaccharides (NSPs), a fraction of dietary fiber, are present in the plant cell wall and are considered antinutritional factors as they affect diet digestibility, given that poultry and pigs lack endogenous enzymes capable of breaking them down (Campestrini et al. 2005). In this context, the use of exogenous enzymes can enhance nutrient utilization by improving digestibility.

The use of multi-carbohydrases in diets can reduce the antinutritional effects of NSPs, especially when arabinofuranosidases (ABFs) are present. These debranching enzymes work synergistically with xylanases to break down the complex fibrous structures of plant cell walls. By removing branches from fibrous complexes, ABFs create access points for xylanases to reach the main chain of fiber structures, further enhancing their efficiency in breaking down the fiber in the cell wall. As a result, nutrients initially trapped within the cell wall are released, making them available for endogenous enzyme action and improving overall feed digestibility (Cozannet et al. 2017). The use of multi-carbohydrases to enhance the degradation and absorption efficiency of fiber-rich diets can be a key tool for the cost-effective development of the swine industry. Therefore, this study evaluated the effects of including a multi-carbohydrase complex (MCC) in corn-based and corn–HP-DDGS-based diets on nutrient digestibility and energy metabolizability in growing pigs (35 to 85 kg).

## Materials and methods

All procedures involving animal management were conducted in accordance with the regulations approved by the Ethics Committee on Animal Use of the Federal University of Minas Gerais (UFMG—CEUA), Brazil, under Protocol CEUA: 254/2020.

### Animals and experimental procedure

The experiment was carried out in the swine metabolism and digestibility laboratory of the Swine Production Sector (NEPSUI) at the Institute of Agricultural Sciences, Federal University of Minas Gerais (UFMG/ICA). A total of eight castrated male pigs, genetically homogeneous from different litters but of the same commercial genetic line (Topigs Norsvin TN70 * Talent^®^), were used. The pigs had an average initial body weight of 35 kg (approximately 75 days of age). Animals were assigned to 4 dietary treatments using a replicated 4 × 4 Latin square design, and each pig was considered the experimental unit.

Each experimental period lasted 17 days and consisted of a 5-day adaptation to the experimental diets in individual slatted-floor pens (1 m × 1 m), followed by 5 days of adaptation to the metabolic cages, and a subsequent 7-day total collection phase for feces and urine in the metabolic cages. The metabolic cages were adjusted to the size of the animals and housed in a temperature-controlled room. After the completion of each collection period, the animals were fasted for 24 h before being reassigned to a different dietary treatment, ensuring that no pig received the same diet in consecutive periods. The overall experimental period lasted 71 days.

The treatments were as follows: a control diet (corn/soybean meal) with or without MCC, and a test diet (20% HP-DDGS in replacement to corn and soybean meal) with or without MCC. MCC was added at a rate of 50 g ton^−1^ (Rovabio^®^ Advance, Adisseo, France), primarily composed of xylanases and arabinofuranosidases, along with other carbohydrases. Experimental diets were formulated using the nutritional model and recommendations provided in the Topigs Norsvin Manual (2019) for growing pigs. The ingredient composition of the experimental diets is shown in Table 1, while the chemical composition of HP-DDGS is detailed in Table 2.

**Table 1:** Composition of the experimental diets (as-fed basis).

Ingredients	Control	Control + MCC^a^	HP-DDGS^b^	HP-DDGS + MCC
**Corn (7.8%)**	63.69	63.69	48.78	48.78
**Soybean Meal (46%)**	33.18	33.18	28.16	28.16
**DDGS corn (42%)**	0.00	0.00	20.00	20.00
**Limestone**	1.19	1.19	1.20	1.20
**Dicalcium phosphate**	0.92	0.92	0.98	0.98
**Sodium chloride**	0.37	0.37	0.37	0.37
**L-Lysine HCL**	0.18	0.18	0.20	0.20
**L-Threonine**	0.07	0.07	0.00	0.00
**DL-Methionine**	0.08	0.08	0.00	0.00
**L-Tryptophan**	0.02	0.02	0.00	0.00
**Celite 1%**	0.10	0.10	0.10	0.10
**Starch**	0.05	0.00	0.05	0.00
**MCC**	0.00	0.01	0.00	0.01
**Mineral Premix^c^**	0.10	0.10	0.10	0.10
**Vitamin Premix^d^**	0.05	0.05	0.05	0.05
**Total**	100.00	100.00	100.00	100.00
**Calculated composition**				
**Crude protein, %**	19.84	19.84	24.79	24.79
**Crude fiber, %**	3.40	3.40	3.88	3.88
**Ether extract, %**	2.92	2.92	4.47	4.47
**Neutral detergent fiber, %**	10.73	10.73	15.06	15.06
**Acid detergent fiber, %**	4.12	4.12	5.66	5.66
**ME, Mcal/kg**	3206.00	3206.00	3168.00	3168.00
**NE, Mcal/kg**	2341.00	2341.00	2313.00	2313.00
**Total calcium, %**	0.82	0.82	0.82	0.82
**Digestible phosphorus, %**	0.22	0.22	0.22	0.22
**SID Lysine, %**	1.06	1.06	1.06	1.06
**SID Methionine, %**	0.37	0.37	0.40	0.40
**SID Met + Cys, %**	0.66	0.66	0.76	0.76
**SID Threonine, %**	0.71	0.71	0.75	0.75
**SID Tryptophan, %**	0.21	0.21	0.21	0.21
**SID Arginine, %**	1.21	1.21	1.32	1.32
**SID Valine, %**	0.82	0.82	1.01	1.01
**Sodium, %**	0.35	0.35	0.35	0.35
Analyzed composition				
**Dry matter, %**	89.64	89.68	90.27	90.40
**Crude protein, %**	22.47	21.56	27.13	27.02
**Ether extract, %**	3.70	3.35	5.88	5.81
**Acid detergent fiber, %**	4.61	4.38	6.44	6.79
**Neutral detergent fiber, %**	10.62	10.31	15.97	15.93
**Gross energy, kcal/kg**	3902.00	3845.00	4138.00	4117.00
**Ash, %**	5.48	5.21	4.89	4.87
**Calcium, %**	0.79	0.75	0.72	0.70
**Phosphorus, %**	0.62	0.57	0.60	0.60
**Acid-insoluble ash, %**	0.20	0.26	0.28	0.29

a MCC = multi-carbohydrase complex.

b HP-DDGS = high-protein distillers dried grains with solubles.

c Copper sulfate (13.00 g/kg), Iron Sulfate (100.00 g/kg), Manganese Monoxide (50.00 g/kg), Sodium Selenite (184.00 mg/kg), Zinc Sulfate (95.00 g/kg), Calcium Iodate (1000 mg/kg).

d Vitamin A (225,00000 UI/kg), Vitamin D3 (380,0000 IU/kg), Vitamin E (200,000 IU/kg), Vitamin K (10,000 mg/kg), Biotin (1,000 mg/kg), Folic Acid (9,000 mg/kg), Niacin (120,000 mg/kg), Pantothenic Acid (60,000 mg/kg), Vitamin B2 (20,000 mg/kg), Vitamin B1 (8,000 mg/kg), Vitamin B6 (12,000 mg/kg), and Vitamin B12 (100,000 mcg/kg).

**Table 2:** Analyzed nutrient composition of ingredients (as-fed basis).

Item	HP-DDGS
**Dry matter, %**	92.43
**Crude protein, %**	42.16
**Ether extract, %**	12.28
**Ash, %**	2.58
**Organic matter, %**	89.85
**Neutral detergent fiber, %**	32.43
**Acid detergent fiber, %**	11.49
**Crude fiber, %**	5.54
**Calcium, %**	0.02
**Phosphorus, %**	0.42
**Sodium, %**	0.02
**Potassium, %**	0.50
**Magnesium, %**	0.12
**Chloride, %**	0.20
**Sulfur, %**	0.50
**Gross energy, kcal/kg**	3732.00
**Net energy, kcal/kg**	2342.00
**Lysine, %**	1.05
**Methionine, %**	0.97
**Methionine + cysteine, %**	1.81
**Threonine, %**	1.54
**Tryptophan, %**	0.32
**Isoleucine, %**	1.62
**Arginine, %**	1.88
**Valine, %**	2.13
**Leucine, %**	5.05
**Phenylalanine, %**	2.19
**Histidine, %**	1.06
**Glycine, %**	1.53
**Cystine, %**	0.84
**Tyrosine, %**	1.78
**Proline, %**	3.40
**Glutamic acid, %**	7.67
**Serine, %**	1.96
**Alanine, %**	3.03

The diets were provided according to metabolic body weight (BW^0,60^) determined by weighing the animal at the start of each period. The amount of feed was calculated based on maintenance energy x metabolic weight (kg^0,60^) × production factor (3 × maintenance; Steinet al. 2006)/dietary energy density:


$$\text{DM}\;\text{intake}\;\text{kg}/d={179\;\text{kcal}\;\text{NE}\times \text{BWkg}}^{0,60}\times 3,0\;\left(\text{PF}\right)/2320\;\text{kcal}\;\text{NE}/\text{kg}$$

The value of 179 kcal represents the maintenance energy requirement for growing pigs, according to Noblet et al. (1999) and Barea et al. (2010). The value of 2320 kcal NE/kg corresponds to the average net energy content of the experimental diets.

Inside the facility, a data logger was placed at the animals’ mid-height to monitor temperature and humidity throughout the experimental period.

### Metabolizability and digestibility trial

The metabolizability and digestibility trial was conducted using the total collection method for feces and urine across four collection periods, with each treatment consisting of 8 repetitions, where each animal was considered an experimental unit. Each period lasted 17 days, during which the animals underwent a 10-day adaptation period to the diets (5 days in individual pens and 5 days in metabolic cages) followed by 7 days of total feces and urine collection (in metabolic cages).

For urine collection, pigs were housed in metabolism cages that allowed total and separate collection of feces and urine. To prevent nitrogen losses and microbial degradation, 25 mL of 6 N hydrochloric acid were added to each urine collection bucket. Feces and urine were collected twice daily at 8 am and 5 pm. The total daily output of feces and urine was weighed and recorded, and a 5% subsample of each daily collection was retained and stored at −20°C for subsequent analyses. At the end of each 7-day collection period, daily subsamples were homogenized, pooled by animal within period, and prepared for chemical analysis.

After the completion of a collection period, the animals were fasted for 24 hours and then redistributed among treatments, ensuring that the same animal did not receive the same diet as in the previous period. Following this redistribution, the animals were fed for 10 days for adaptation and then underwent another 7-day collection period.

### Laboratory analyses

The experimental diets and feces were analyzed for moisture, crude protein, and gross energy. Additionally, gross energy was also analyzed in urine. The results were used to calculate dry matter intake, apparent dry matter digestibility coefficient, apparent gross energy digestibility coefficient, energy metabolization coefficient, apparent crude protein digestibility coefficient, and apparent digestible and metabolizable energy values (Matterson et al. 1965).

### Statistical analyses

Data were statistically analyzed using the Proc GLM procedure in SAS (SAS Inst. Inc., Cary, NC), with each animal considered an experimental unit. The four treatments were compared using initial weight as a covariate, and an analysis of covariance was conducted, incorporating cereal source, enzyme inclusion, animal, and period as main effects. Mean comparisons were performed using the Student-Newman-Keuls test, with an alpha value of 0.05 set to determine the level of significance between means.

## Results

The average temperature and relative humidity recorded during the experimental period were 23.6 ± 2.47°C and 74 ± 18%, respectively. No interactions were observed between diets with or without HP-DDGS and enzyme use for the variables studied (*P* > 0.10).

### Effect of ingredients

The results are presented in Table 3. The inclusion of HP-DDGS significantly reduced dry matter (DM) intake (*P* < 0.001) and increased fecal output (*P* < 0.001) compared to diets without HP-DDGS. The results also showed that, compared to corn, the use of HP-DDGS reduced the apparent total tract digestibility (ATTD) coefficients of DM by 5.23 percentage points and crude protein (CP) by 5.11 percentage points (*P* < 0.001) and decreased the gross energy (GE) digestibility and the energy metabolizability coefficient (EMC) by 7.8 and 8.39 percentage points, respectively (*P* < 0.001). Diets containing HP-DDGS showed a reduction of 158 kcal/kg in digestible energy (DE) (*P* = .005) and 189 kcal/kg in metabolizable energy (ME) (*P* < 0.001) compared to the control diet.

**Table 3:** Effects of multi-carbohydrase complex (MCC) supplementation in corn–soybean meal or corn–HP-DDGS–based diets on dry matter intake, fecal output, and apparent total-tract digestibility (ATTD) coefficients of dry matter (DM), crude protein (CP), gross energy (GE), digestible energy (DE), metabolizable energy (ME), and energy metabolizability coefficient (EMC) in growing pigs (35–85 kg).

Variable	Control	Control	HP-DDGS	HP-DDGS	Cereal		MCC		Coeff Var	RSD^b^	R²	*P*-value^a^		
		+ MCC		+ MCC	Control	HP-DDGS	Without	With				Cereal	MCC	Cereal × MCC
**DM Intake, g**	7168	7946	6897	7124	7557	7011	7033	7535	6.95	19.47	92.28	< 0.001	0.435	0.332
**Fecal output, g**	1498	1506	1828	1957	1502	1892	1663	1731	11.98	26.64	83.71	< 0.001	0.653	0.562
**ATTD of DM %**	78.86	81.05	73.1	76.34	79.95	74.72	75.98	78.70	4.35	7.23	69.93	< 0.001	0.002	0.366
**ATTD of CP %**	78.07	79.99	73.36	74.48	79.04	73.93	75.72	77.24	0.657	6.23	79.36	< 0.001	< 0.001	0.493
**ATTD of GE %**	79.34	81.05	71.71	73.07	80.19	72.39	75.53	77.06	0.468	7.37	89.54	< 0.001	0.003	0.412
**EMC %**	76.63	78.91	68.83	69.94	77.77	69.38	72.73	74.43	0.471	8.23	89.77	< 0.001	0.002	0.815
**DE kcal/kg**	3428	3502	3276	3339	3465	3307	3352	3421	0.470	5.81	98.43	0.005	0.005	0.079
**ME kcal/kg**	3310	3409	3145	3196	3359	3170	3228	3303	0.472	6.64	98.42	< 0.001	0.051	0.153

a Values with *P* < 0.05 are considered statistically significant. Analyses consider the main effects of cereal source (corn–soybean meal or corn–HP-DDGS–soybean meal), enzyme inclusion (with or without MCC), and their interaction (Cereal × MCC).

b Residual standard deviation (RSD). *n* = 8 pigs per treatment.

c MCC = multi-carbohydrase complex.

d HP-DDGS = high-protein distillers dried grains with solubles.

### Effect of enzyme

The results are presented in Table 3. Multi-carbohydrase complex inclusion had no effect on DM intake or total fecal output. However, its supplementation improved the ATTD of DM by 2.72% (*P* = 0.002) and the CP by 1.52 percentage points (*P* < 0.001). It also enhanced the GE digestibility by 1.53 percentage points (*P* = 0.003) and the EMC by 1.7 percentage points (*P* = 0.002). Additionally, MCC increased DE by 69 kcal/kg (*P* = 0.005) and ME by 75 kcal/kg (*P* = 0.051).

## Discussion

The present study demonstrated that animals fed HP-DDGS exhibited lower feed intake and higher fecal output, resulting in a reduced ATTD of DM compared to the control diet. This indicates that a larger portion of the ingested feed was not utilized by the animal. The decreased nutrient utilization with HP-DDGS was further reflected in the lower ATTD for CP and GE digestibility, along with a decline in the EMC. These findings are consistent with those of McDonnell et al. (2011), who reported a linear decline in DM digestibility when using an ethanol co-product, as DDGS levels increased (0 to 30%) in finishing pigs. This suggests that high DDGS inclusion in swine diets can reduce nutrient digestibility.

More recent studies have also reported variability in energy and amino acid digestibility among different HP-DDGS sources, emphasizing that nutrient utilization is highly dependent on processing technology and chemical composition (Cristobal et al. 2020; Rho et al. 2017). Therefore, the reductions observed in the present study likely reflect both the intrinsic fiber concentration and the physicochemical characteristics of the evaluated HP-DDGS source, reinforcing the importance of ingredient characterization when incorporating ethanol co-products into swine diets.

The reduced digestibility observed with HP-DDGS can primarily be attributed to its elevated fiber concentration and the limited capacity of pigs to degrade NSPs. Recent studies have demonstrated that fiber digestibility in pigs is less than 20% in the small intestine and below 50% across the entire gastrointestinal tract (Jha & Berrocoso 2015). This is because pigs lack the necessary digestive enzymes or produce them in insufficient quantities to effectively degrade NSPs (Gutierrez et al. 2013). As a result, the low fiber digestibility of HP-DDGS may reduce nutrient utilization and lead to increased excretion of organic matter by these animals.

The lower GE digestibility and EMC observed in the HP-DDGS diets in this study resulted in a decrease in their DE and ME. This could also be linked to the higher concentration of insoluble fiber (Gutierrez et al. 2013). Additionally, the reduction in these energy values may be explained by the metabolizable energy/digestible energy ratio described by Noblet and Perez (1993), which suggests that protein levels reduce this ratio by 2% for every 1% increase in CP in the diet. Given that HP-DDGS is rich in protein, this leads to greater energy loss in these formulations. Higher dietary protein concentrations are also associated with increased urinary nitrogen excretion and greater heat increment, which may contribute to lower net energy efficiency in high-protein diets (Noblet et al. 1994).

In this context, nutritional strategies aimed at improving fiber degradation may help mitigate the limitations associated with high-fiber co-products.

However, the results of this experiment show that the use of MCC can be a strategy for feeding growing and finishing pigs, increasing the digestibility of diets, including those with higher fiber content, such as HP-DDGS diets. Specifically, the addition of MCC improved the ATTD of DM, CP, and GE, as well as the DE and ME values, regardless of HP-DDGS inclusion. This highlights MCC’s ability to optimize overall feed utilization. These improvements demonstrate the potential of targeted enzyme supplementation to enhance nutrient availability in both conventional and co-product-based diets.

The MCC used in this study is rich in xylanase and arabinofuranosidases, debranching enzymes that target arabinoxylans, the main fraction of NSPs in corn and corn HP-DDGS (Jaworski et al. 2015; Ward 2021). These enzymes break down NSPs in the plant cell wall, releasing nutrients that were previously inaccessible, which allows endogenous enzymes to further improve nutrient digestibility (Cozannet et al. 2017). Arabinofuranosidase plays a critical role in removing arabinose side chains from the xylan backbone, thereby facilitating xylanase access to the core structure and enhancing overall fiber degradation efficiency. This synergistic mode of action likely explains the positive responses observed in the present study.

Saleh et al. (2019) observed a positive effect from the inclusion of carbohydrases (xylanase + arabinofuranosidase) in broiler diets, resulting in improved growth performance and nutrient digestibility. Similarly, Cozannet et al. (2017) reported that supplementation with a multi-carbohydrase complex in broiler diets improved nutrient digestibility by an average of 3.9%. In swine, Chen et al. (2023) demonstrated that supplementation with a multi-carbohydrase complex significantly increased the digestibility coefficients of GE and CP, supporting the positive effects observed in the present study.

In contrast, Boucher et al. (2021) evaluated two sources of DDGS, including a novel source with reduced fiber concentration, and reported that the inclusion of a multi-carbohydrase enzyme blend (xylanase, glucanase, cellulase, amylase, invertase, and protease) did not improve energy digestibility in growing pigs. The lack of response observed in that study may be related to differences in enzyme profile and substrate specificity. The compatibility between the enzymatic composition and the structural characteristics of the fibrous substrate appears to be a key factor determining the efficacy of enzyme supplementation. This discrepancy reinforces that enzyme efficacy depends not only on ingredient composition but also on the match between enzyme profile and substrate structure.

While enzyme responses may differ among species, pigs derive a proportionally greater contribution of released energy from fiber degradation compared with poultry. In broilers fed DDGS, only 2.1% and 4.6% of the metabolizable energy released by MCC originated from soluble and insoluble fiber degradation, respectively, whereas in swine approximately 36% of the enzymatically released energy is attributed to fiber hydrolysis (Cozannet et al. 2017). This contrast highlights the greater relevance of carbohydrase supplementation in pig diets containing fibrous ingredients. Therefore, the magnitude of response observed in the present study is biologically plausible considering the greater capacity of pigs to utilize energy released from fiber degradation.

Although corn is generally a highly digestible ingredient with low fiber content, the inclusion of MCC can still promote significant improvements in the digestive process, highlighting the importance of enzyme use in conventional diets. For diets containing HP-DDGS, the addition of MCC yielded even more notable improvements, as it facilitated greater degradation of fibrous substrates, allowing the enzymes to act more effectively. This reduced the antinutritional effects of the diet and increased nutrient availability for the animal.

These findings suggest that enzyme supplementation may provide greater nutritional and potentially economic benefits when fiber-rich co-products are included at higher dietary levels.

In conclusion, the use of HP-DDGS in pig diets offers a cost-effective alternative while providing an adequate nutritional value. Moreover, the inclusion of MCC further enhances diet efficiency, nutrient and energy digestibility, in a corn/soy diet or in a corn/soy/HP-DDGS, by improving overall feed utilization.

## Abbreviations

(ATTD): Apparent total tract digestibility
(CP): Crude protein
(DE): Digestible energy
(DM): Dry matter
(EMC): Energy metabolizability coefficient
(GE): Gross energy
(HP-DDGS): High-protein corn dried distillers’ grains with solubles
(ME): Metabolizable energy
(MCC): Multi-carbohydrase complex
(NSP): Non-starch polysaccharides
